# Of love and other demons: depicting human rabies in Colombia

**DOI:** 10.1016/j.heliyon.2022.e09703

**Published:** 2022-06-10

**Authors:** Luis Octavio Tierradentro-García, María Camila Cortés-Albornoz, Claudia Talero-Gutiérrez

**Affiliations:** a Department of Radiology, Children's Hospital of Philadelphia, Philadelphia, PA, USA; b Neuroscience Research Group (NeURos), Escuela de Medicina y Ciencias de La Salud, Universidad Del Rosario, Bogotá D.C., Colombia; c Centro de Neurociencia Neurovitae-UR, Escuela de Medicina y Ciencias de La Salud, Universidad Del Rosario, Bogotá D.C., Colombia

**Keywords:** Rabies, Human rabies, Epidemiology, Colombia, Literature

## Abstract

Human rabies has been described by various cultures in many countries around the world. Gabriel García Márquez's novel *Of love and other demons* recounts the story of a girl who, in colonial Colombia in the 18^th^ century, was bitten by a rabid dog. This paper aims to review the general status of the disease in Colombia and interweave it with García Márquez's book. Developed countries have successfully controlled dog-mediated rabies virus transmission, whereas in some countries in Latin America wildlife and canine rabies are still an issue. Our approach suggests that although the measures that have been taken to mitigate dog-transmitted rabies have worked well in most parts of the world, greater efforts are required to control sylvatic rabies transmitted by bats or other mammals, as occurs in Colombia. Since developing countries are the most affected by the disease at present, transdisciplinary commitment between human and veterinary sectors is necessary to fight against rabies virus transmission.

## 1 Introduction

In his literary masterpiece *Of love and other demons,* the Colombian writer Gabriel García Márquez, one of the most remarkable exposers of magical realism in Latin America, depicts the tragic story of a girl, known as Sierva María, who was bitten by a rabid dog. In the epoch of 18th-century Cartagena de Indias, Colombia, the lack of scientific knowledge and the Catholic Church's influence over the population made it difficult for the physicians to diagnose it as a case of human rabies or “demonic possession” [1].

Human rabies, which is caused by negative-stranded RNA lyssaviruses (e.g. rabies virus (RABV)) [2], is an acute viral encephalomyelitis and is transmitted by dogs in more than 90% of cases [3]. In Latin America, dog mediated and wildlife rabies is endemic [4]; 14.7% of all cases in the region correspond to *Desmodus rotundus (D. rotundus)* bat transmission, mostly in Peru, followed by Brazil, Mexico, Colombia, Panama, El Salvador, and Nicaragua, representing the second most common cause of RABV transmission [5]*.* In Colombia, multiple cases of cross-species transmission have been detected, for example, from 1993 to 2002 a total of 22 cases were secondary to dog transmission, one case was due to cats, and three cases were due to hematophagous bats transmission [5, 6]. Even today, no cure has been developed, and RABV is still fatal in most cases once the clinical signs appear.

The greatest impact of dog-mediated disease today is seen in developing countries. Prevention and control efforts have been relatively successful in Colombia during the last few decades, but continuing transdisciplinary commitment between the human and veterinary sectors is necessary to mitigate RABV transmission, both in Colombia and worldwide [7]. This paper will contribute to the visibility and further understanding of historical, epidemiological, and clinical aspects of human rabies in Colombia that could promote research and effective control strategies. Our objective is to review the general status of human rabies in Colombia and interweave it with García Márquez's book.

## 2 Methods

We conducted a narrative review search of literature regarding the current status of rabies in Colombia and Latin America, and its strategies for prevention and management. The search terms were: “rabies disease”, “rabies virus”, “human rabies disease”, “rabies in Colombia”, “rabies in Latin America”, “rabies epidemiology”, “rabies prevalence”, “rabies incidence”. The following databases/websites were explored: PubMed, Embase, ScienceDirect, SciELO, LILACS, the World Health Organization (WHO), the Pan American Health Organization (PAHO), the Colombian National Institute of Health, and the Colombian Ministry of Health, among others. We selected papers, published in indexed journals, that addressed relevant epidemiological information in Colombia and Latin America, but also included information reported by governmental institutions such as surveys, bulletins, and epidemiological reports. Since this paper was not conducted as a systematic review, the articles/reports/bulletins were selected subjectively, at the discretion of authors. Other papers were included for expanding the discussion in terms of pathophysiology, clinical presentation, management, and prevention strategies. No Institutional Review Board approval was required for the development of the present article.

## 3 Historical aspects of rabies in the Americas

In the 18^th^ century, rabies was very common throughout Europe and was related to zoonosis in wild foxes, wolves, and dogs [8]. There were also cases of rabid vampire bats in the Americas, which, according to annals written by Spanish narrators, attacked the conquistadors in the Mexican peninsula of Yucatan and the Darien strait (current Colombian territory) [8, 9]. It is noteworthy that before the Spanish conquest, RABV was present in bats and skunks but apparently not yet described in dogs. Canine RABV was introduced into America as dogs were brought from Europe in the 15^th^ century, leading to allelic replacement and genetic admixture of the aboriginal canines [9]. For more than 200 years, the disease went unnoticed, until its outbreak in the 18^th^ century. The first rabies epizootic occurred in Mexico in 1709, and cases of human rabies were documented from 1776 to 1778 in the Greater Antilles [9]. By the 19^th^ century, other countries of South America (e.g., Peru, Argentina, and Chile) had also been affected [9]. Various major epizootics were reported in North America during the 18^th^ and 19^th^ centuries. In addition to rabid dogs, there were also rabid mesocarnivores, including skunks, foxes, coyotes in North America; later, in the 20^th^ century, cases of mongoose rabies were reported in several Caribbean islands. It was not until the 20^th^ century that approaches towards the accurate diagnosis, isolation of the infectious agent, and management strategies for rabies first appeared [8].

## 4 Clinical features of rabies in *Of love and other demons*

As mentioned in *Of love and other demons*, any physical or behavioral change in an individual who had been bitten by a dog was considered an initial symptom of rabies disease, as concern about the development of the disease was widespread in the population of Cartagena de Indias, and a lack of awareness about the precise physical manifestations associated with rabies led to panic. Three months after being bitten by the rabid dog, Sierva María had not had any symptoms but was already imprisoned in a convent [1].

Human rabies disease is characterized by five clinical stages from the incubation period to death. According to Udow et al., the overall mean incubation period is 54 days, but with an average of 64 days for dog bites and 51 days for bat bites; although in rare cases, incubation periods can be greater than several years [10, 11, 12]. Average survival is usually between one and two weeks from clinical onset, yet rare cases have lasted more than one month [3, 13]; furthermore, location of the bite is related to prognosis, as also stated in the book: *“The wound was far from the area of greatest risk, and no one recalled any bleeding. The most probable outcome was that Sierva María would not contract rabies”* [1]. Human rabies can manifest clinically as furious (also known as encephalitic) or classic rabies, which is present in more than two-thirds of patients, and paralytic rabies, which resembles Guillain-Barré syndrome [3].

Prodromal clinical features are common to both forms of rabies, including local neuropathic pain in a third of patients. The clinical manifestations of furious rabies include hypersalivation, irritability, agitation, hyperesthesia, fluctuation of the state of consciousness, hydrophobia, aerophobia, spasms, autonomic signs [14], and sensory deficits including loss of pinprick sensation as well as loss of proprioception. Physical examination may note hypertonic muscles and hyperreflexia with abnormal plantar response, while flaccid weakness with areflexia only appears in the comatose stage [3]. Differential diagnoses for furious rabies include botulism, diphtheria, intoxication with phenothiazines and amphetamines, delirium tremens, and *Datura fastuosa* ingestion [15, 16].

The paralytic form shows motor manifestations, incontinence, and myoedema. Cardinal motor symptoms are lower motor neuron ascending weakness and fasciculations, which can progress to coma [3]. The differential diagnoses for the paralytic form include Guillain-Barré syndrome, polio, and herpesvirus B infection [15, 16]. Progression to coma and death occurs more rapidly in paralytic rabies than in furious rabies [17].

In *Of love and other demons*, a patient with alleged rabies was locked up in a hospital in Cartagena de Indias; specifically, *“in the pavilion that housed raving lunatics”.* He was described as *“…an old mulatto with a beard and hair like cotton. By now half his body was paralyzed, but the disease had endowed the other half with so much strength that he had to be tied to keep him from smashing himself to pieces against the walls* [1]*.”* These symptoms resemble the characteristics of the paralytic manifestation of rabies.

## 5 Current epidemiological status of rabies

Rabies is an under-reported, neglected zoonosis with the highest fatality rate of any human infectious disease. Several attempts to defeat human rabies, such as animal control programs, were first implemented in the 20^th^ century [9]. In 2020, 80 countries (47%) reported no human deaths from rabies, and is estimated an increase in zero death tendency through 2023, 2025, and 2030. It is predicted that 155 countries (92%) are expected to achieve this goal [18]. The risk of contracting a RABV infection is 5–80% per bite [16], and despite being a vaccine-preventable viral disease, one death related to rabies occurs every 9 min around the world [19]. However, despite the high lethality rate of human rabies, some cases of patients surviving the infection have been reported [20, 21, 22, 23, 24].

The most devastating impacts of human rabies on public health are the high mortality rate and loss of economic productivity from premature death. India has become the country with the highest incidence of human rabies, accounting for over 35% of the global rabies burden [25]. As the disease mostly occurs in low-income countries, within communities with poor access to the health system and lack of laboraroty-based surveillance, most victims die without being admitted to hospitals or receiving palliative care [26], which contributes to the under-recording of rabies-related deaths worldwide. The WHO and other international organizations are coordinating efforts to mitigate dog-transmitted rabies globally by 2030 [27,28]. According to the latest Rabies Global Conference (held in Geneva, Switzerland, in 2015), the main programs for eliminating human rabies should be focused on the mass vaccination of dogs, better access to pre- and post-exposure prophylaxis for high-risk populations, and improvements in surveillance and public awareness [29]. In an attempt to raise awareness, an international campaign coordinated by the Global Alliance for Rabies Control commemorates World Rabies Day on September 28, every year [30] **.**

## 6 Rabies in Latin America

The burden of human rabies in the Americas is small, especially when compared with that of endemic countries in Africa or Asia, and studies account for 130 to 200 deaths per year, with most estimated rabies cases in Haiti [25, 31]. In the last 5 years, at least nine Latin American countries have reported deaths related to human rabies. In 2020 alone, there were 12 cases of human rabies disease in Latin America: two cases in Brazil, one case in Colombia, three cases in Cuba, one case in Mexico, two cases in Peru, and one case in the Dominican Republic [32, 33].

Rabies can be epidemiologically classified as urban or sylvatic rabies. Although dog-transmitted human rabies has decreased in frequency, bats are emerging as important vectors of transmission to humans [6]. Curiously, RABV transmitted by bats only occurs in the Americas [34], where, between 2005 and 2013, there were 122 cases of human rabies transmitted by hematophagous bats, with most cases reported in Peru (n = 62), followed by Brazil (n = 46) [35]. Between 2000 and 2017 in Brazil, there were 188 cases of human rabies in total [36].

Mammalian lyssavirus reservoirs in Latin America primarily include dogs, bats, crab-eating foxes, and hoary foxes [37]; although, in Brazil, non-human primates such as marmosets have reportedly transmitted RABV to humans [38]. Countries like Chile, Brazil, and Argentina have made efforts to identify wild rabies reservoirs (mainly hematophagous and insectivorous bats, which may affect livestock) [39, 40]. *D. rotundus* seems to be the only Latin American hematophagous bat that acts as a RABV reservoir [6]. Diaz et al. conducted a study in which they identified eight distinct antigenic types among 288 rabies samples from 17 Latin American and Caribbean countries. Two of these forms were associated with enzootic disease in dogs and vampire bats [41]. Unlike some Asian countries, RABV transmission due to dog slaughtering is not common in Colombia or other Latin American countries [42].

Potent canine rabies vaccines were key factors in the fight against rabies in the 20^th^ century. In the mid-1950s, a live-attenuated vaccine was created in Mexico by Fuenzalida and Palacios. During the next two decades, an extremely large number of dogs were vaccinated; nevertheless, there was no decrease in rabies cases [43]. In 1983, the member countries of the Pan American Health Organization (PAHO) committed to implementing coordinated actions to fight rabies [43]. Massive dog vaccination schemes using a new inactivated cell-culture-derived vaccine started in 1989, and the prevalence of canine rabies decreased from more than 3000 in 1990 to 70 in 2007 [44]. Since then, Latin American countries have seen a decline of 95% in the prevalence of human rabies [45]. However, economic, social, and cultural disparities in some peripherical areas threaten the success of major rabies-related health politics in Latin America [43, 46].

The most recent large-scale study into human rabies transmitted by dogs in Latin America was published in 2018 and included data from 21 countries collated from 1998 to 2014 [47]. However, several smaller studies in individual countries have also been published [48, 49, 50, 51, 52, 53, 54, 55, 56, 57, 58, 59, 60]. From 2010 to 2012, there were 40 human rabies cases transmitted by dogs and 63 cases transmitted by bats, and the areas with the highest incidences were Haiti, Bolivia, Guatemala, Dominican Republic, Brazil, and Peru [61, 62]. According to Velasco-Villa et al., there were 139 human cases reported in Latin America from 2005 to 2015, with the highest prevalence rates in Haiti and Bolivia [43]. Between 2010 and 2014, there were just a few cases of human deaths from rabies reported in Bolivia, Brazil, the Dominican Republic, Guatemala, Haiti, and Peru; however, the various data sources are inconsistent [63].

Viral typing is important to accurately assess the effectiveness of control efforts in enzootic dog-maintained RABV transmission [43]; however, laboratory-based confirmation techniques are not used by some South American countries, including Uruguay and Paraguay. Poor surveillance is also a feature of several Central American countries, e.g., Panama, Costa Rica, Nicaragua, and Belize [32, 43]. As a consequence of under-reporting, there is a lack of accurate data on the current state of rabies in Latin America. Maxwell et al. compiled the information provided by the Ministries of Health and Agriculture from 31 Latin American countries in 2015, and they highlighted rabies as a priority endemic zoonosis along with leptospirosis and brucellosis [64].

Some countries have relaxed their control measures against rabies viral transmission (e.g., by suspending mass canine vaccination and obtaining fewer doses of post-exposure prophylaxes), which may increase the rate of rabies in dogs and, potentially, the rate of transmission to humans [9, 45, 65]. At the time of writing this manuscript, there is an ongoing rabies epidemic in Arequipa, Peru, due to a decrease in dog vaccination coverage, decreased surveillance, and increased survival times of infected canines [66]. The concomitant COVID-19 pandemic poses even more obstacles to those attempting to control this event, which could be explained by the obvious priority given to COVID-19. Moreover, 25% of rabies endemic countries had reassigned surveillance staff to COVID-19 efforts [67]. Overall, COVID-19 pandemic could increase awareness of the existing health disparities in terms of neglected zoonosis and the importance of lifeguarding animal health, continuing dog vaccination, and post-exposure vaccine access to maintain rabies control all over the world [67].

## 7 Rabies in Colombia

The study of rabies as a public health issue in Colombia started in the 1950s, and a decade later, high-impact campaigns against rabies were implemented in several cities [68, 69, 70]. The average national canine immunization coverage from 1994 to 2005 fluctuated between 45% and 63% [71].

In Colombia, health professionals must notify the “Sistema Nacional de Vigilancia en Salud Pública” (SIVIGILA) when an individual has been attacked by a potential rabies-virus-transmitting animal, and it is mandatory to report confirmed cases in animals and humans. The Colombian National Health Institute (Instituto Nacional de Salud) oversees the accurate reporting of location, number, depth of lesions, and vaccination history in humans, as well as the animal vaccination background, the clinical state of the animal, and the place where the event occurred. In the end, every case is categorized according to the probability of viral transmission as “no-risk”, “low-risk”, or “high-risk” exposure. This classification allows appropriate management to be implemented in every case [72].

As in other Latin American countries, Colombia is committed to the elimination of canine rabies. Efforts to this end are implemented by several governmental organizations [35], each of which focuses on a specific target, such as the identification of potentially rabid animals (especially dogs and cats), laboratory tests on dead animals, and surveillance of wild reservoirs. According to Cifuentes-Jiménez et al., the two most influential RABV reservoirs and vectors in Colombia are dogs and *D. rotundus* [6].

In Colombia, cross-species transmission can occur, e.g., dog–dog, dog–fox–dog, dog–cat, bat–cat, skunk–cat, cat-other small wild mammals [46]. Between 2004 and 2019, there were 86 confirmed rabies cases in domestic animals (dogs and cats), most of which were identified on the Caribbean Coast (more than 76%). Two cases of canine rabies were reported in the Magdalena region and one bat-related case in the Cundinamarca region of Colombia during the first 6 months of 2019. These areas represent high-risk locations for viral circulation and transmission to humans and animals. Fifty-six foci of infection were also recorded, with an important focal point on the north coast [73], and relevant interventions have been carried out in these regions to characterize and control RABV through updated surveillance protocols [74]. It should be stressed hematophagous bats are not the only bat reservoirs of RABV; two species of frugivorous bats were recently identified as RABV reservoirs in Colombia [75]. However, since any bat species may be potentially infected, all bat exposures should receive proper attention.

Rabies surveillance of human infections in Colombia focuses on evaluating the expositional risk of RABV transmission by animals [76, 77]. As an exemplary event, seventeen cases were reported in Chocó in 2004–2005, a low-income, rural, and humid region prone to sylvatic transmission [78]. Characterization of human rabies in Colombia from 2007 to 2011 showed that the prevalence of rabies viral transmission was higher in young adult males [79].

Between 2000 and 2017, there have been 38 cases of human rabies in Colombia, all of which were fatal, and most were transmitted by bats [35, 73]. The highest occurrence was on the Pacific Coast (53% of cases), followed by the Caribbean Coast (18%), and the Oriental and Central regions (13% each). The two most recent cases of mortality related to human rabies occurred in Huila, a dry, tropical region located in the Andean region of South Colombia. The first case (2020), a 26-year-old female was bitten by a cat that was infected by a viral variant from a hematophagous bat. Three weeks after the bite, she showed clinical signs and symptoms, and died 1 month after exposure [80]. The second case (2021) was a 29-year-old male who was bitten in his face by a cat. Three weeks after the bite he started complaining of malaise, headaches and muscle spasms, and was hospitalized; the patient died two and a half weeks after hospitalization [81]. The viral characterization of the cases (by antigenic variant) in Colombia included V1 (dog), V3 (hematophagous bat), V4 (insectivorous bat), V8 (skunk), and atypical V1 [35].

**Figure 1:**
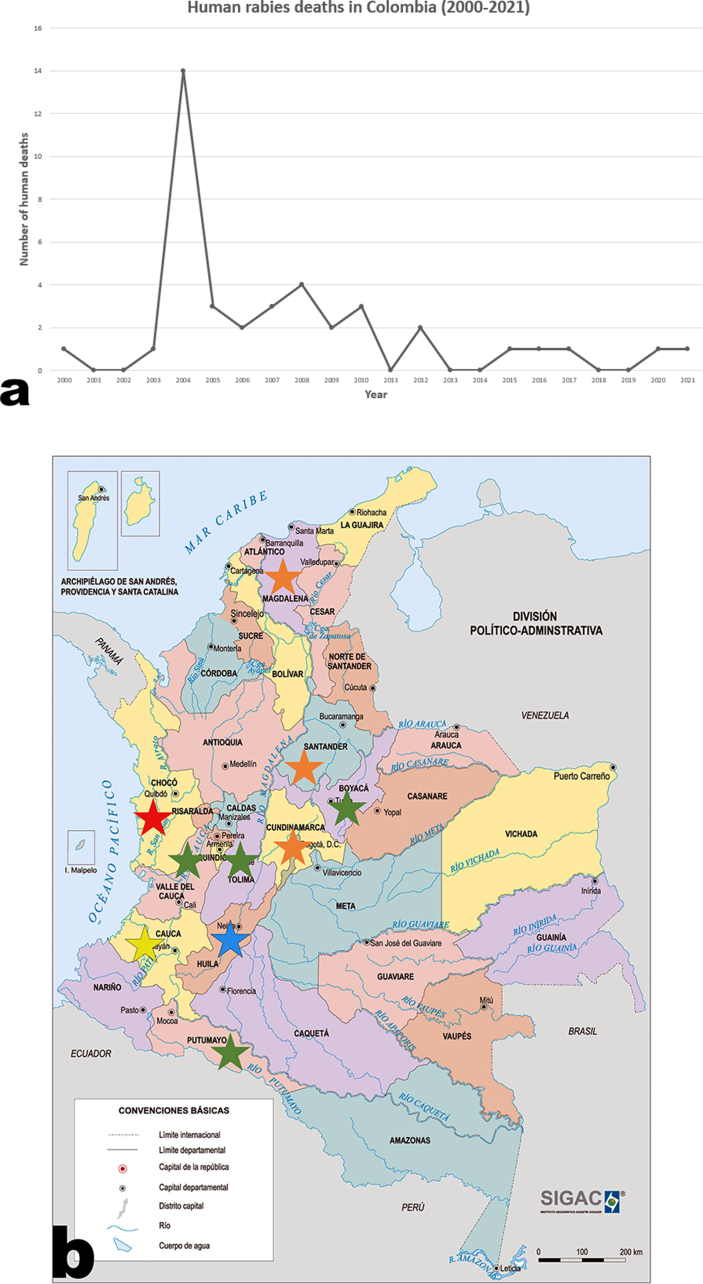
**Human rabies-related deaths in Colombia.** (a) Graph represents cases of human rabies-related deaths over 21 years; note the peak in 2004. (b) Topographical map depicts number of cases per region. Red star = 14 cases (Chocó); orange stars = 4 cases (Magdalena, Santander, and Cundinamarca); yellow star = 3 cases (Cauca); blue star = 2 cases (Huila); green stars = 1 case (Boyacá, Casanare, Tolima, Putumayo, and Valle del Cauca). Of note, the map reveals that most cases occurred in the mountainous region and near the Pacific coast. Modified from https://geoportal.igac.gov.co/contenido/mapas-nacionales.

While surveillance is mandated by governmental authorities, underreporting may still occur in communities of rural areas (e.g., Orinoquia and Amazon regions, in Eastern and Southern Colombia, respectively), which are more prone to be infected by a rabid bat. These regions deserve a focused plan for the detection and prevention of sylvatic rabies. Table 1 summarizes the human rabies cases recorded in Colombia from 2000 to the present day, and a map of the affected regions is represented in Figure 1.

**Table 1:** Human deaths from rabies in Colombia in the current century†.

Year	Regions [number deaths]	Number of deaths	Vector [number of deaths]
**2000**	Putumayo [1]	1	Dog [1]
**2003**	Cundinamarca [1]	1	Cat [1]
**2004**	Chocó [14]	14	Bat [14]
**2005**	Chocó [3]	3	Dog [3]
**2006**	Magdalena [2]	2	Cat [2]
**2007**	Magdalena [2], Casanare [1]	3	Cat [2], bat [1]
**2008**	Cauca [3], Santander [1]	4	Cat [1], bat [3]
**2009**	Boyacá [1], Santander [1]	2	Cat [2]
**2010**	Tolima [1], Santander [2]	3	Cat [2], bat [1]
**2012**	Valle del Cauca [2]	2	Cat [2]
**2015**	Cundinamarca [2]	1	Cat [1]
**2016**	Cundinamarca [1]	1	Cat [1]
**2017**	Cundinamarca [1]	1	Cat [1]
**2020**	Huila [1]	1	Cat [1]
**2021**	Huila [1]	1	Cat [1]

† Data collected until April 2022. Table modified from Sánchez, M. del P., Díaz Sanchez, O. A., Sanmiguel, R. A., Ramirez, A. A., & Escobar, L. (2019). Rabia en las Américas, varios desafíos y « Una Sola Salud: Artículo de revisión. Revista de Investigaciones Veterinarias Del Perú, 30 (4), 1361–1381.

## 8 Advances in prevention and management strategies

As mentioned in *Of love and other demons*, an ash-gray dog bit Sierva María on her left ankle, but apparently, no one was alarmed. Her maid *“treated the bite herself with lemon and sulfur, and washed the bloodstain from the girl's petticoat…”.* After 2 days, her mother was told of the incident with the dog. *“Since the maid had not told her where the bite was located, Bernarda –her mother– raised the girl's chemise and examined her inch by inch, using the light to follow the penitential braid that curled around her body like a lion's tail. At last, she found it: a little break in the skin on her left ankle, with a scab of dried blood and some almost invisible abrasions on the heel”* [1].

All dogs that were suspected of having rabies, based on observable clinical signs, were killed and hung in a visible place. This allowed the people of the area to note their appearance and be aware of the risks if someone had been bitten by the same dog [1, 8]. In the book, Sierva María's mother *“…was so certain of her reasoning that she did not mention the matter to her husband or think about it again until the following Sunday when the maid went to the market alone and saw the carcass of a dog that had been hung from an almond tree to let everyone know it had died of rabies. One glance was all she needed to recognize the blaze on the forehead and the ash-gray coat of the dog that had bitten Sierva María. But Bernarda was not concerned when she heard the news. There was no reason to be: The wound was dry and not even a trace of the abrasions remained”* [1].

García-Marquez's narrative describes several approaches to treat rabies that were used during the 18th century [1]. Primitive strategies that were used to cure rabies consisted of eating a cockerel's brain, applying grease and honey as a poultice over the wound, or even eating the flesh of a mad dog [8]. Sierva María's father was so concerned that he consulted all physicians, pharmacists, barber-surgeons, and magical healers. *“A young physician from Salamanca opened Sierva María's closed wound and applied caustic poultices to draw out the rank humors. Another attempted to achieve the same end with leeches on her back. A barber-surgeon bathed the wound in her own urine, and another had her drink it. At the end of two weeks, she had been subjected to two herbal baths and two emollient enemas a day and was brought to the brink of death with potions of natural antimony and other fatal concoctions. The fever subsided, but no one dared proclaim that rabies had been averted. Sierva María felt as if she were dying. At first, she had resisted with her pride intact, but after two fruitless weeks she had a fiery ulcer on her ankle, her body was scalded by mustard plasters and blistering poultices, and the skin on her stomach was raw. She had suffered everything: vertigo, convulsions, spasms, deliriums, looseness of the bowels and bladder, and she rolled on the floor howling in pain and fury. Even the boldest healers left her to her fate, convinced she was mad or possessed by demons”* [1]*.*

Injuries from animal bites are common occurrences in emergency rooms around the world [82]. Cleansing and vigorously washing the wound with abundant water and soap remains the first strategy after a dog bite, and virucidal agents (e.g., povidone-iodine, hydrogen peroxide, alcohol) can also be applied [19]. Currently, the cornerstone of rabies prevention in humans consists of controlling transmission from dogs [15, 16]; according to the WHO, vaccinating 70% of dogs in high-risk areas would break the RABV transmission cycle [19]. Veterinarians led the first national program to vaccinate dogs in Japan in 1921, and most industrialized countries followed suit by vaccinating dogs at risk of rabies in urban areas. However, the prevalence of rabies in wildlife has increased in several regions, such as some countries of the Middle East [83]. Vaccination of dogs and wild carnivores in the Americas has increased effective immunization, recognizing the importance of wildlife vaccination, and bat population management in the region [84]. However, we acknowledge this is an issue in Colombia, where most human cases of rabies were transmitted by cats, that were infected by rabid bats. Several efforts have been done to fight wildlife rabies as well, including the application of oral vaccination (i.e. vaccine-laden baits) for wild mammals such as raccoons, foxes, and coyotes. Regarding bats, current questionable management practices include the use of oral anticoagulants (vampiricides) or capture/killing of bats and destruction of their roosts. For instance, Costa Rican authorities started implementing specific measures to face RABV transmission to livestock and humans by vampire bats, in order to reduce farmers' economic losses and human transmission [85]. Similar considerations should be applied by governmental and local public health authorities in Colombia to avoid transmission to domestic animals and humans. Novel research on oronasal and topical vaccines against rabies in bats seems promising, although we acknowledge that, given the huge variety of bat species, it is challenging to obtain a wide coverage with these strategies [84, 86, 87].

Active and passive immunization can be applied in the context of exposure to RABV. In 2018, the WHO updated its position on rabies immunization strategies according to exposure categories [88, 89]. There are pre-exposure and post-exposure vaccination schemes. Pre-exposure vaccination is encouraged in individuals, such as veterinarians, spelunkers, animal handlers, certain travelers, and laboratory personnel, who are at a high risk of contact with wildlife rabies reservoirs. In the case of post-exposure vaccination, it is applied in the deltoid region in a 4-dose scheme) [89, 90]. Table 2 summarizes the latest WHO recommendations on rabies prophylaxis. In addition, post-exposure prophylaxis is an important measure to consider after contact with a suspect animal, although these biologics are not affordable or scarce in some regions. Management comprises post-exposure vaccination and a single dose of rabies immunoglobulin for all transdermal exposure. In individuals that have been previously vaccinated, after exposure, two vaccine boosters are administered on days 0 and 3 [91]. Major advances have been made to increase the accessibility of the vaccine and facilitate its application; for instance, the intradermal route of administration is non-inferior in terms of efficacy and much more cost-effective than the intramuscular injection [29, 92].

**Table 2:** Current World Health Organization recommendations on rabies prophylaxis.

WHO 2018 recommendations on rabies prophylaxis				
	Exposure classification	Immediate action	Immunization schedules	Vaccine administration route
**Post-exposure prophylaxis**	Category I	Immunologically naive individuals: Wash exposed skin	None.	Intramuscular (IM) or intradermal (ID) route - Adults and Children older than 2 years old: administration in the deltoid region - Children younger than 2 years old: administration in the anterolateral thigh or suprascapular region.
	Category II	Wash exposed skin and vaccinate	**Immunologically naive individuals (one of the options):** - 2-sites ID on days 0, 3 and 7. - 1-site IM on days 0, 3, 7, and between days 14–28 - 2-sites IM on days 0 and 1-site IM on days 7, 21 **Previously immunized individuals (one of the options): *Except if already received complete PEP within the previous* 3 months** - 1-site ID on days 0 and 3 - at 4-sites ID on day 0 - at 1-site IM on days 0 and 3	
	Category III	Wash exposed skin, vaccinate and Rabies Immune Globulin administration if available (within 7 days from the start of vaccination)	**Immunologically naive individuals (one of the options):** - 2-sites ID on days 0, 3 and 7 - 1-site IM on days 0, 3, 7, and between days 14–28 - 2-sites IM on days 0 and 1 site IM on days 7, 21 **Previously immunized individuals (one of the options): *Except if already received complete PEP within the previous* 3 months** - 1-site ID on days 0 and 3 - at 4-sites ID on day 0 - at 1-site IM on days 0 and 3	
**Pre-exposure prophylaxis**	None, In all individuals with occupational high risk	Vaccination	**Immunologically naive individuals (one of the options):** - 2 sites ID vaccine administration on days 0 and 7 - 1-site IM vaccine administration on days 0 and 7 **Immunodeficient individuals:** Previous schedule vaccination plus a third vaccine between days 21–28	

WHO: World Health Organization; ID: intradermal; IM: intramuscular; PEP: post-exposure prophylaxis.

Presently, pharmacological approaches to human rabies disease include benzodiazepines, antipsychotics, and analgesics to alleviate suffering in patients. Nevertheless, no clear benefits, in terms of survival, have been seen in patients given these measures, and most of them are considered palliative. The most recent version of the Milwaukee protocol for the management of rabies disease states that coma induction is not recommended [3, 93]. Other strategies proposed in past versions of the protocol, such as barbiturates, nimodipine, and ribavirin, are no longer used. Aggressive supportive care combined with immunotherapy, neuroprotection, and antiviral therapies may be useful in specific cases [94]. In Colombia, the National Institute of Health developed an exposure-based management algorithm and protocol. The risk of transmission is classified as no exposure, mild exposure, or severe exposure, also known as category I, II, and III according to the WHO exposure categories [89, 95]. Except for large wounds, suturing is not generally recommended [96]. After vigorous washing, physicians must use tetanus toxoid and antibiotics and fill out the epidemiological notification form [95]. In case of mild exposure, the rabies vaccine should be formulated according to a four dose scheme on days 0-0-7-21 [97]. On the other hand, severe exposure is considered as any injury caused by an unknown domestic animal (dog or cat), or a domestic animal with rabies signs during a 10-day window. In this case, the 4-dose rabies vaccine (except if post-exposure prophylaxis was received in the 3 months previous) and a single dose of rabies immunoglobulin should be administered. Rabies immune globulin heterologous (40 IU/kg) or homologous (20 IU/kg) serums are available in all national territories, and are also considered in the WHO Essential Medicine list for post-exposure prophylaxis [95, 98].

## 9 Conclusions

The work of the Nobel prize-winner Gabriel García Márquez, author of *love and other demons,* illustrates how some public health issues remain tangible despite multiple efforts to end them. In the modern world, human rabies is highly preventable. To achieve this, mass vaccination of all domestic animals at risk of exposure and standardization of vaccine procurement are required to widen coverage in rural areas and developing countries. At present, apart from a few remarkable cases, clinically declared cases of human rabies are incurable.

Records on rabies prevalence or incidence are not always easily accessed or updated in real-time. When they are available, the data on rabies are not always shared between human and animal health sectors. Notifying officials of rabies cases in dogs or bites from potential RABV transmitters, including bats, should be obligatory. In a One Health context, multiple collaborative efforts involving doctors, veterinarians, scientists, vaccine developers, and governmental institutions, as well the implementation of community engagement approaches, are needed to make progress in eliminating the risk of rabies to humans.

At the end of Garcia Marquez's novel, Sierva María is condemned to live in isolation in a convent. In those same halls, a priest falls in love with her, and they start to secretly meet. Pitifully, he is sent to a leprosy hospital, and she is left behind, awaiting her exorcism. It is uncertain whether she actually had rabies; however, since several weeks/months had passed after she was bitten and no typical clinical manifestations were present, it was concluded that “*As for Sierva María, after so many weeks it did not seem probable that she would contract the disease. The only risk at present was that she, like so many others, would die of the cruelty of the exorcism”* [1].

## Declarations

### Author contribution statement

Luis Octavio Tierradentro-García: Conceived and designed the experiments; Performed the experiments; Analyzed and interpreted the data; Contributed reagents, materials, analysis tools or data; Wrote the paper.

María Camila Cortés-Albornoz: Analyzed and interpreted the data; Contributed reagents, materials, analysis tools or data; Wrote the paper.

Claudia Talero-Gutiérrez: Conceived and designed the experiments; Analyzed and interpreted the data; Contributed reagents, materials, analysis tools or data; Wrote the paper.

### Funding statement

This research did not receive any specific grant from funding agencies in the public, commercial, or not-for-profit sectors.

### Data availability statement

No data was used for the research described in the article.

### Declaration of interests statement

The authors declare no conflict of interest.

### Additional information

No additional information is available for this paper.
